# CoDe: a web-based tool for codon deoptimization

**DOI:** 10.1093/bioadv/vbac102

**Published:** 2023-01-02

**Authors:** Divya Sharma, Tracey Baas, Aitor Nogales, Luis Martinez-Sobrido, M Michael Gromiha

**Affiliations:** Protein Bioinformatics Lab, Department of Biotechnology, Indian Institute of Technology Madras, Chennai 600036, India; Texas Biomedical Research Institute, San Antonio, TX 78227, USA; Center for Animal Health Research, CISA-INIA-CSIC, Madrid 28130, Spain; Texas Biomedical Research Institute, San Antonio, TX 78227, USA; Protein Bioinformatics Lab, Department of Biotechnology, Indian Institute of Technology Madras, Chennai 600036, India

## Abstract

**Summary:**

We have developed a web-based tool, CoDe (Codon Deoptimization) that deoptimizes genetic sequences based on different codon usage bias, ultimately reducing expression of the corresponding protein. The tool could also deoptimize the sequence for a specific region and/or selected amino acid(s). Moreover, CoDe can highlight sites targeted by restriction enzymes in the wild-type and codon-deoptimized sequences. Importantly, our web-based tool has a user-friendly interface with flexible options to download results.

**Availability and implementation:**

The web-based tool CoDe is freely available at https://web.iitm.ac.in/bioinfo2/codeop/landing_page.html.

**Supplementary information:**

Supplementary data are available at *Bioinformatics Advances* online.

## 1 Introduction

Codons play a critical role in protein synthesis by constituting the genetic code that governs the process of translating information contained in a DNA or RNA sequence to the corresponding protein sequence (Gouy and Gautier, 1982; Parvathy *et al.*, 2022; Quax *et al.*, 2015). There are 64 codon combinations but only 20 amino acids to encode. The genetic code is considered degenerate because 18 of 20 amino acids may be encoded by multiple synonymous codons, with the exception of tryptophan (W) and methionine (M) that are encoded by a single codon (Athey *et al.*, 2017; Gouy and Gautier, 1982; Parvathy *et al.*, 2022). Importantly, different organisms exhibit preferential usage of certain codons over others, and this biological phenomenon is termed codon usage bias (Athey *et al.*, 2017; Gouy and Gautier, 1982; Quax *et al.*, 2015; Sharp and Li, 1987). Although the reasons underlying the existence of such codon usage bias are not well known, codon selection does have a significant impact on many cellular processes, including transcription, mRNA stability, protein expression efficiency and accuracy, among others (Athey *et al.*, 2017; Lavner and Kotlar, 2005; Liu, 2020; Parvathy *et al.*, 2022; Quax *et al.*, 2015; Wu *et al.*, 2019).

It has been observed that highly expressed proteins are commonly encoded by genes with optimal codon usage (Frumkin *et al.*, 2018; Sharp and Li, 1987). Therefore, codon usage bias has been applied for codon optimization or codon deoptimization strategies to enhance or reduce, respectively, gene expression in different systems and/or organisms such as mammalian, bacteria or insect cells (Athey *et al.*, 2017; Kwon *et al.*, 2016; Sharp and Li, 1987). Codon optimization requires that the peptide sequence is encoded by the most frequent codons and this method has been widely used for biotechnological, pharmaceutical or research purposes (Gary and Weiner, 2020; Gustafsson *et al.*, 2012; Nieuwkoop *et al.*, 2020; Sethi *et al.*, 2021; To and Cho, 2021). On the other hand, codon deoptimization is achieved by replacing original codons for less frequent codons. Codon deoptimization potential has recently begun to be explored and exploited to generate recombinant RNA or DNA viruses as live-attenuated vaccines and/or vaccine vectors (Baker *et al.*, 2015; Burns *et al.*, 2006; Cai *et al.*, 2020; Cheng *et al.*, 2015, 2017; Lorenzo *et al.*, 2022; Meng *et al.*, 2014; Nogales *et al.*, 2014). Importantly, in both cases, codon optimization and deoptimization modifications at the nucleotide or codon level do not affect the amino acid sequence of the proteins or their functionality and/or immunogenicity (Athey *et al.*, 2017; Gouy and Gautier, 1982; Lavner and Kotlar, 2005; Parvathy *et al.*, 2022; Sharp and Li, 1987).

There are multiple bioinformatics tools and webservers allowing codon optimization of input sequences facilitated by codon bias usage that can be chosen from several organisms. However, no equivalent exists for codon deoptimization, and researchers need to perform codon deoptimization manually, increasing the chance for errors and variability in the output sequences and data. Moreover, the lack of a web-based tool for codon deoptimization impedes broad application of the new methodology for biotechnological and research applications, including the high potential for vaccine development and molecular biology. Therefore, the development of codon deoptimization software is highly desired and its demand is expected to increase (Baker *et al.*, 2015; Gustafsson *et al.*, 2012; Liu, 2020; Meng *et al.*, 2014; Nieuwkoop *et al.*, 2020; Quax *et al.*, 2015; Wu *et al.*, 2019).

In this work, we have developed a webserver, CoDe, which is a codon deoptimization tool capable of selecting the most suitable codon for DNA or RNA sequences based on codon usage tables of several organisms. The tool provides a codon-deoptimized nucleotide sequence as output and highlights the amino acids translated by changed codons. We have designed the tool to include multiple options, including different codon usage tables, codon deoptimization of specific regions of a gene, codon deoptimization of selected amino acids, or the identification of restriction enzyme sites in the wild-type or codon-deoptimized sequences. The webserver is freely available at https://web.iitm.ac.in/bioinfo2/codeop/landing_page.html.

## 2 Server description

**Fig. 1.**
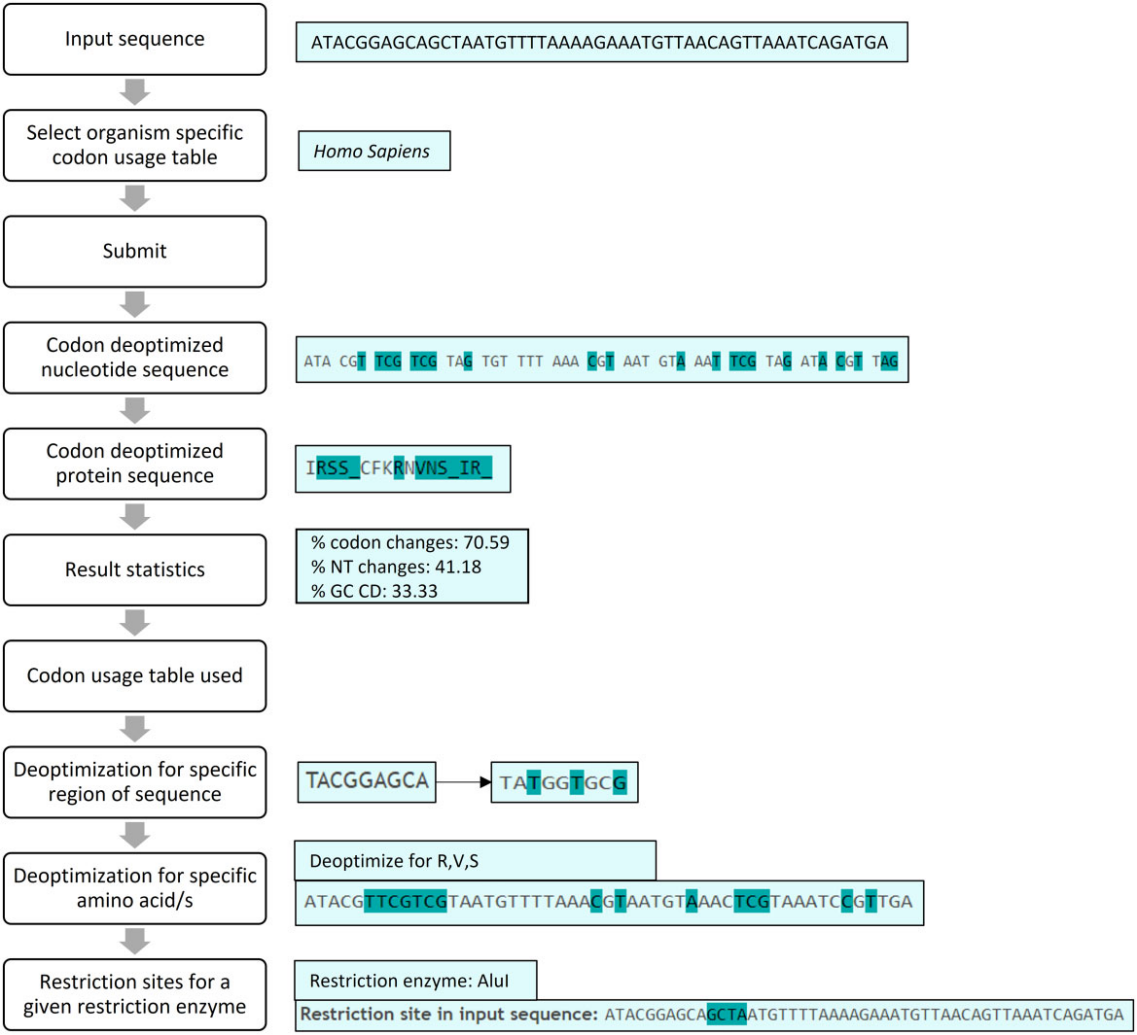
Flowchart of CoDe functionalities with an example of input nucleotide sequence using *Homo sapiens* codon usage table. The nucleotides and codons highlighted in cyan were changed to codon deoptimized in the input sequence

We have created a user-friendly webserver for codon deoptimization of DNA or RNA sequences. The different functionalities of CoDe are outlined in Figure 1 and described here. For further information, we have included a tutorial sequence set and also a tutorial video at https://web.iitm.ac.in/bioinfo2/codeop/landing_page.html, describing all functionalities of CoDe.

### 2.1 Codon deoptimization for a given DNA/RNA sequence

The CoDe webserver computes the codon-deoptimized sequence from the given DNA or RNA sequence, using the codon usage bias among organisms and compares the codon frequencies coding for the same amino acid. The lowest frequency codon encoding the same amino acid is chosen as the deoptimized codon. CoDe offers organism-specific codon usage tables for nine species that can be selected from the input page: *Homo sapiens* (Supplementary Table S1), *Macaca mulatta*, *Mus musculus*, *Rattus norvegicus*, *Gallus*, *Arabidopsis thaliana*, *Drosophila melanogaster*, *Saccharomyces cerevisiae* and *Escherichia coli.* The codon usage tables have been generated from the widely used ‘Kazusa’ database (Nakamura *et al.*, 2000). Output data include codon-deoptimized nucleotide sequences, result statistics and the codon usage table selected for codon deoptimization of the given DNA or RNA sequence. The result statistics include the number of codons in the given sequence, number and percentage of codon changes after deoptimization, the total number of nucleotides in the given DNA or RNA sequence, number and percentage of nucleotide changes after codon deoptimization and percentage of GC content within the input and output (i.e. codon-deoptimized) sequences.

### 2.2 Codon deoptimization of a specific nucleotide region and/or amino acid(s)

CoDe has an option to deoptimize the sequence based on specific region and/or amino acid(s). For deoptimizing specific regions of the input DNA or RNA sequence, users will need to enter the nucleotide positions (i.e. a number) to be deoptimized based on the sequence. For deoptimizing specific amino acid(s) in the given DNA or RNA sequence, users will need to indicate the amino acid(s) to be codon deoptimized by using single-letter amino acid code(s), separated by commas.

### 2.3 Identification of restriction sites for enzymes

Users can also view restriction enzyme sites within the input and the codon-deoptimized sequences by specifying the restriction enzyme name in usual naming convention.

**Fig. 2.**
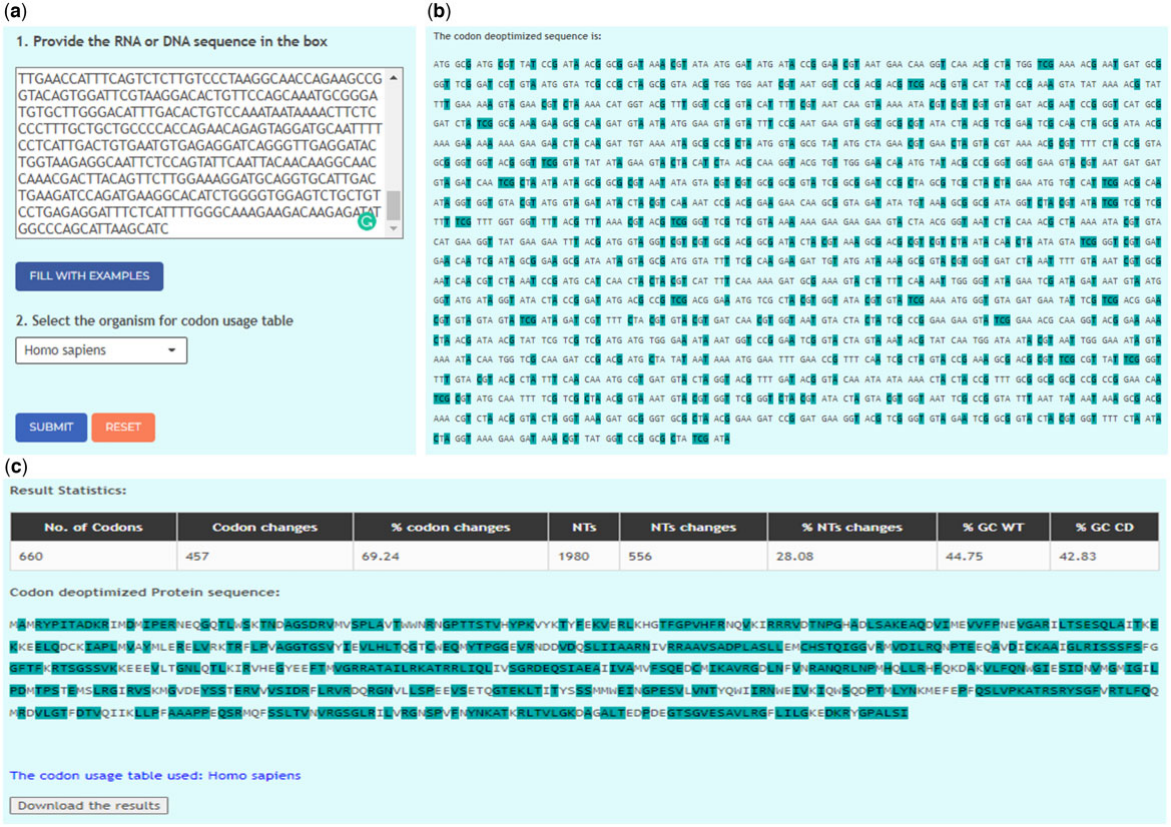
(**a**) Input nucleotide sequence (1) and selection of organism for codon usage table (2); (**b**) output for codon deoptimized sequence with color-coded deoptimized nucleotides; and (**c**) result statistics and protein sequence where amino acids with color-coded deoptimized codons.

Figure 2 shows an example to deoptimize the input nucleotide sequence.

Steps:

1. Copy and paste the nucleotide sequence into the input box. Select the organism-specific codon usage table and click the ‘Submit’ button (Fig. 2a).
2. The output page shows the deoptimized nucleotide sequence (Fig. 2b).
3. The output page also shows result statistics and amino acids that have been codon deoptimized in the protein sequence.
4. In addition, the output page shows the codon usage table that has been used to codon deoptimize the sequence. This table can be downloaded by clicking on ‘The codon usage table used’. The results from the codon deoptimization could also be downloaded by clicking on the ‘Download the results’ button (Fig. 2c).

Examples for additional features are provided in the Supplementary Material.

## 3 Implementation

The scripts to calculate the codon-deoptimized DNA or RNA sequences and the restriction enzyme sites have been written in Python. The Python-CGI scripts are used to render the HTML web pages. The CoDe webserver works with manually typing or copy and paste to input the nucleotide DNA or RNA sequence. The output can be viewed as an html page, as well as downloaded in the text and html formats.

## 4 Unique features

To our knowledge, CoDe is the only webserver that deoptimizes DNA or RNA nucleotide sequences based on codon usage tables from different organisms. A comparison of CoDe with Codon_tools (a python package for codon deoptimization, which is based on CpG count) is provided in Supplementary Table S2. It can also provide partial codon deoptimization of specific regions and/or codon deoptimization of specific amino acid(s). In addition, CoDe provides restriction enzyme sites in wild-type and deoptimized sequences for all restriction enzymes using Biopython’s Restriction package. The codon-deoptimized nucleotides, amino acids, and restriction enzyme sites are color coded for better visibility. The server can deoptimize a 2000-base-long nucleotide sequence in 1.35 s. CoDe shows an accuracy of 100% when compared with the results of manual codon deoptimization (and the same codon usage tables) from previous articles (Baker *et al.*, 2015; Cai *et al.*, 2020; Cheng *et al.*, 2015, 2017; Lorenzo *et al.*, 2022; Nogales *et al.*, 2014).

## 5 Applications

Given the impact of codon usage bias on gene expression, cellular function or viral fitness (Athey *et al.*, 2017; Lavner and Kotlar, 2005; Liu, 2020; Parvathy *et al.*, 2022; Quax *et al.*, 2015), codon deoptimization is a promising biotechnological tool for a broad range of biological research recombinant gene applications, and, most notably, vaccine development.

Vaccination represents the best option to protect humans and animals against infection with viral and bacterial pathogens; therefore, robust strategies to improve the effectiveness of current or novel vaccines is critical to ensure the well-being of human and animal health, as well as the global economy. Protein synthesis from a specific gene can be selectively downregulated by adjusting gene codon usage through deoptimization. This molecular strategy, together with rapid and affordable nucleotide synthesis capabilities, can be used to generate attenuated forms of RNA and DNA viruses that contain gen(es) with deoptimized codons. The attenuated viruses could potentially be used as live-attenuated vaccines, dependent on their safety profile and their ability to induce protective and robust immune responses against challenges with wild-type forms of the virus (Baker *et al.*, 2015; Burns *et al.*, 2006; Cai *et al.*, 2020; Cheng *et al.*, 2015, 2017; Lorenzo *et al.*, 2022; Meng *et al.*, 2014; Nogales *et al.*, 2014). Moreover, viruses with variably recoded genomes could produce a spectrum of attenuation, which would be useful to target specific population groups, such as healthy people, immunocompromised patients, and pregnant women (Baker *et al.*, 2015; Cai *et al.*, 2020; Cheng *et al.*, 2015, 2017; Lorenzo *et al.*, 2022; Nogales *et al.*, 2014). Notably, this technology could also be expanded to protect against other non-viral pathogens, such as bacteria or protozoa.

Currently, codon deoptimization has been a laborious and time-consuming task due to the lack of platforms to automate codon deoptimization. Manual deoptimization by researchers includes the potential for errors and variability. For this reason, we have created CoDe, a user-friendly webserver that will facilitate the effective and rapid codon deoptimization of RNA or DNA sequences. CoDe webserver also provides the possibility of fine-tuning the method to make small adjustments to achieve the desired performance outcome and sequence results. In summary, CoDe opens many possibilities to carry out highly innovative investigations in multiple research fields, and the webserver would provide benefit to a broad range of applications for biotechnology and/or biomedicine.

Although we have included codon usage tables from multiple relevant organisms to deoptimize sequences, other codon usage tables could also be incorporated. Moreover, codon usage tables available in other databases could also be utilized for deoptimization. In future, CoDe can be updated as a software that allow the two possibilities (codon optimization and codon deoptimization) or even combinations of both for the same sequence.

## Supplementary Material

vbac102_Supplementary_Data

## Data Availability

The data and web-based tool CoDe described in this article are available at: https://web.iitm.ac.in/bioinfo2/codeop/landing_page.html.
