# Supplementary material for: CoDe: a web-based tool for codon deoptimization
Source: Bioinform Adv. 2023 Jan 2;3(1):vbac102. doi: 10.1093/bioadv/vbac102 (PMC9832946; doi:10.1093/bioadv/vbac102)
Supplement: vbac102_Supplementary_Data [file vbac102_supplementary_data.docx]

**Supplementary Information**

**CoDe: A web-based tool for Codon Deoptimization**

Divya Sharma^1^, Tracey L. Baas^2^, Aitor Nogales^3,*^, Luis Martinez-Sobrido^2,*^, and M Michael Gromiha^1,*^

^1^ Protein Bioinformatics Lab, Indian Institute of Technology, Madras, Chennai-600036, Tamil Nadu, India.

^2^ Texas Biomedical Research Institute, San Antonio, Texas, 78227, USA

^3^ Center for Animal Health Research, CISA-INIA-CSIC, 28130 Valdeolmos, Madrid, Spain.

.

^*^To whom correspondence should be addressed,

**Contact:** [gromiha@iitm.ac.in](mailto:gromiha@iitm.ac.in) (MMG); [nogales.aitor@inia.csic.es](mailto:nogales.aitor@inia.csic.es) (AN); [LMartinez@txbiomed.org](mailto:LMartinez@txbiomed.org) (LM)

**Examples for additional functionalities of CoDe**

1. **Codon deoptimization for a specific protein region and/or amino acid(s)**

**Figure S1** shows an example to deoptimize the region “50-500” of the nucleotide sequence.

*Steps:*

1. Copy and paste the nucleotide sequence in the input box. Select the organism for codon usage table and click the “Submit” button **(Fig S1a)**.
2. Type “50-500” in “Enter region of the input sequence to deoptimize” box at the next page and click “Submit” **(Fig S1b).**
3. The output page shows deoptimized nucleotide sequence, result statistics and deoptimized protein sequence for the selected region. The codon usage table could be downloaded by clicking on “The codon usage table used” **(Fig S1c)**.
4. **Codon deoptimization of specific amino acid(s)**

**Figure S2** shows an example to deoptimize the nucleotide sequence based on amino acid(s).

*Steps:*

1. Copy and paste the nucleotide sequence in the input box. Select the organism for codon usage table and click the “Submit” button **(Fig S2a)**.
2. Type amino acid(s) to codon deoptimize separated with a coma and using the single code nomenclature (e.g. “R, V, S”) in the “Enter the amino acid(s) to deoptimize” box at the next page and click “Submit” **(Fig S2b).**
3. The output page shows deoptimized nucleotide sequence **(Fig S2c)**, codon deoptimized protein sequence and the number of amino acids and codons deoptimized **(Fig S2d)**.
4. **Finding restriction sites for a given restriction enzyme**

**Figure S3** shows an example to find restriction sites in the input and codon deoptimized sequences for the restriction enzyme EcoRI.

*Steps:*

1. Copy and paste the nucleotide sequence in the input box. Select the organism for codon usage table and click the “Submit” button **(Fig S3a)**.
2. Type the name of the restriction enzyme using standard nomenclature (e.g. “EcoRI”) in “Enter the restriction enzyme to indicate the restriction sites” box at the next page and click “Submit” **(Fig S3b).**
3. The output page shows highlighted restriction sites in the input sequence and in the codon deoptimized sequence, if any. It also shows the sequence of the restriction site and the position of the restriction site in the sequence. If there are no restriction sites for the given restriction enzyme, the output will say “There are no restriction sites for the given restriction enzyme” **(Fig S3c & Fig S3d)**.

**Figures and Tables**


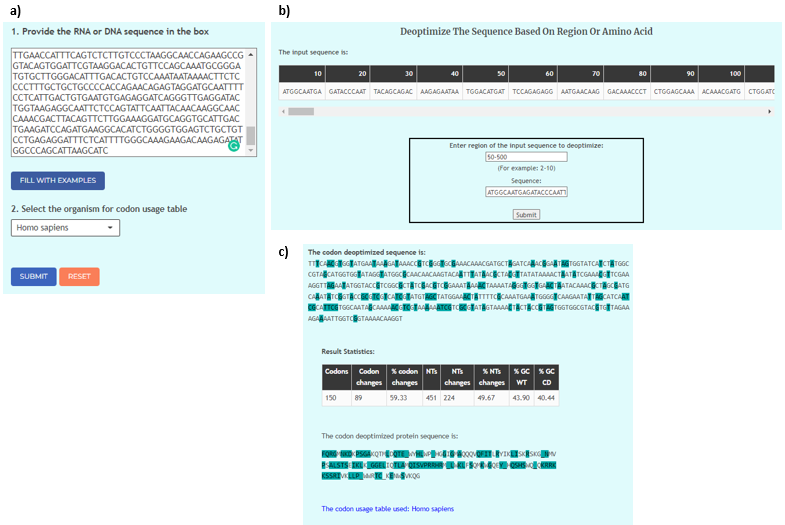


**Fig S1.** A) Input nucleotide sequence (1) and selection of organism for codon usage table (2); b) Select the region of the sequence to codon deoptimize; c) Output for codon deoptimized sequence with colored deoptimized nucleotides, result statistics and deoptimized protein sequence with colored deoptimized amino acids.


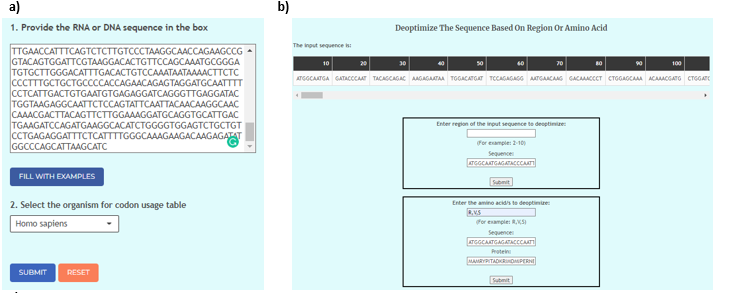


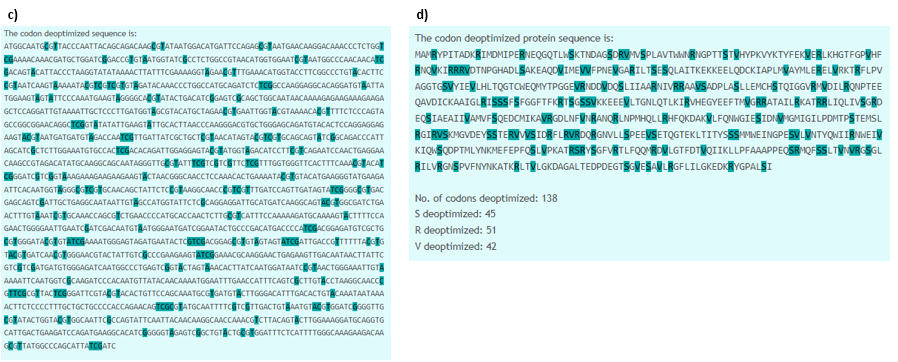


**Fig S2.** a) Input nucleotide sequence (1) and selection of organism for codon usage table (2); b) Indicate the amino acid(s) to codon deoptimize; c) Output for codon deoptimized sequence with colored deoptimized nucleotides; d) deoptimized protein sequence with colored deoptimized amino acids and number of amino acids deoptimized.


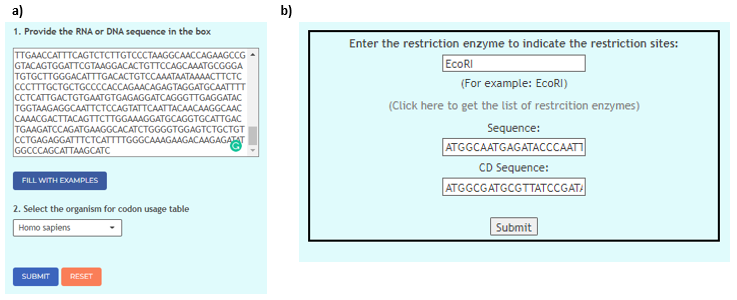


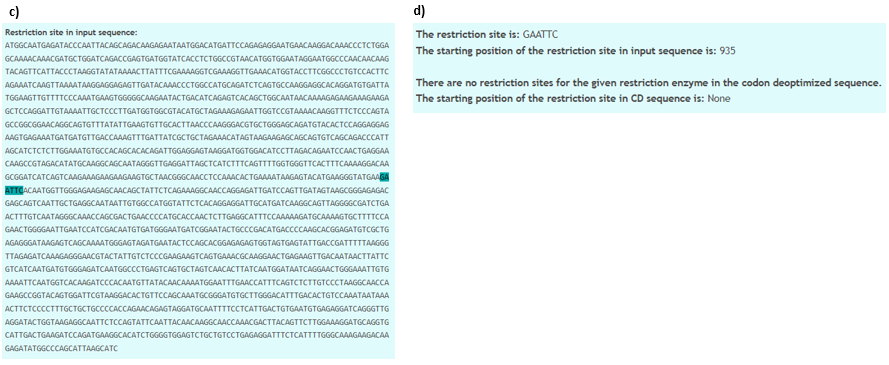


**Fig S3.** a) Input nucleotide sequence (1) and selection of organism for codon usage table (2); b) Introduce the name of the restriction enzyme; c) Output for restriction site in the input sequence; d) Output for restriction site in the codon deoptimized sequence (note that EcoRI is not found in the codon deoptimized sequence).

**Example of *Homo sapiens* codon usage table:**

**Supplementary Table S1. Codon usage table for *Homo sapiens.***

**
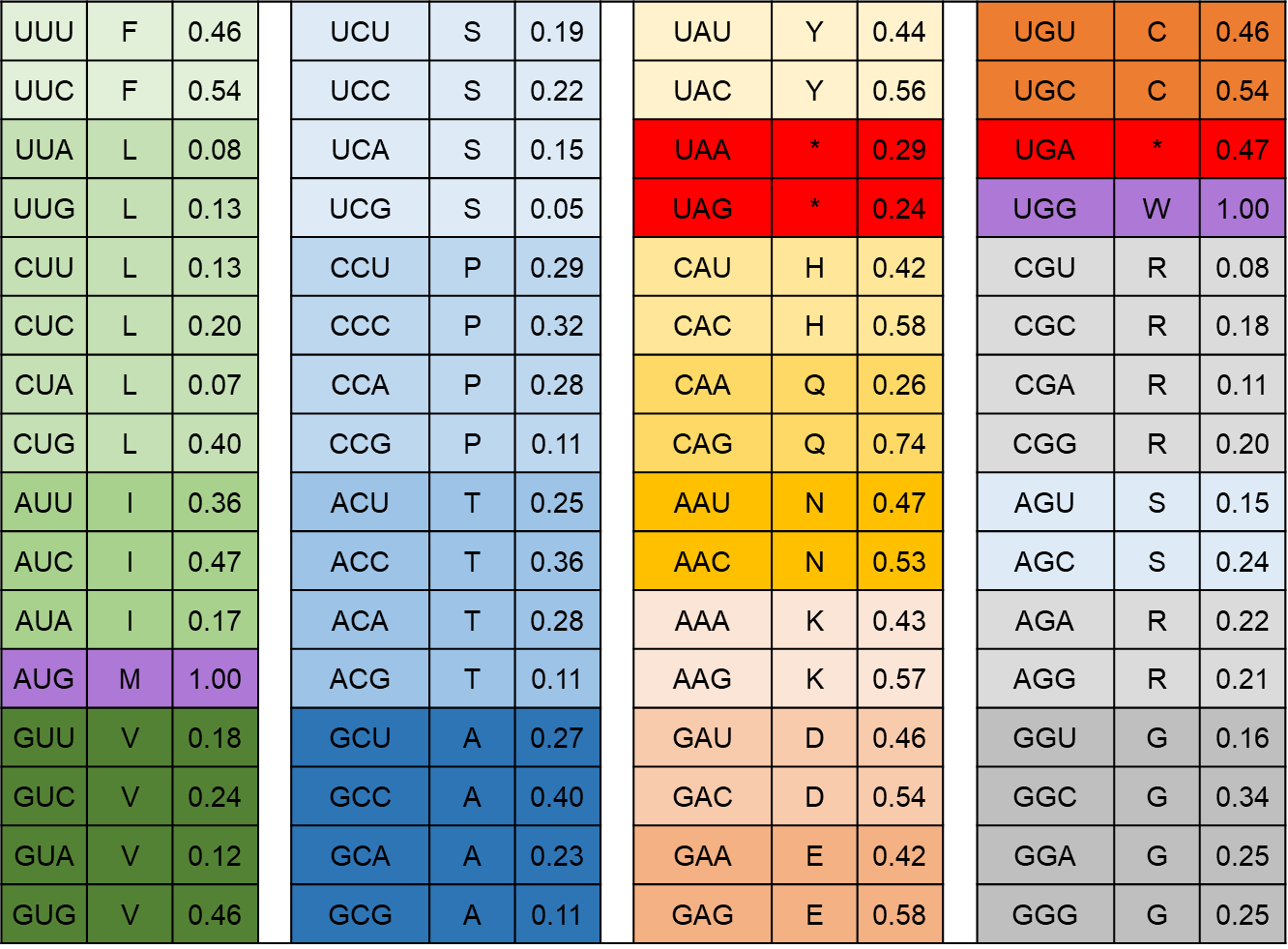
**

Note: A codon chart with the relative synonymous codon usage is displayed. Relative synonymous codon usage fraction is represented. Amino acid residues encoded by a single codon (e.g. M, AUG; and W, TGG) are indicated in purple.

**Supplementary Table S2: Comparison between CoDe and Codon_tools**

| **Criteria** | **CoDe** | **Codon_tools** |
| --- | --- | --- |
| Platform | Web based tool | Python package |
| Working status | Working | Not working, shows import error |
| Criteria used | Based on codon usage bias and multiple codon usage tables | Based on CpG counts |
| Documentation | Tutorial provided | No description/ documentation |
| User-friendly | Easy to use for people with non-programming background and most of researchers in the field of live science. | Not easy to use for people with non-programming background |
| Last update | Latest | Released on Apr 28, 2017, no update |
| Publication status | Published | Not published |
